# Synthetic Host Defense Peptides Inhibit Venezuelan Equine Encephalitis Virus Replication and the Associated Inflammatory Response

**DOI:** 10.1038/s41598-020-77990-3

**Published:** 2020-12-08

**Authors:** Aslaa Ahmed, Allison Bakovic, Kenneth Risner, Stephanie Kortchak, Marcelo Der Torossian Torres, Cesar de la Fuente-Nunez, Timothy Lu, Nishank Bhalla, Aarthi Narayanan

**Affiliations:** 1 National Center for Biodefence and Infectious Disease, Biomedical Research Laboratory, School of Systems Biology, George Mason University, Manassas, VA USA; 2 Machine Biology Group, Departments of Psychiatry and Microbiology, Institute for Biomedical Informatics, Institute for Translational Medicine and Therapeutics, Perelman School of Medicine, Penn Institute for Computational Science, and Department of Bioengineering, University of Pennsylvania, Philadelphia, PA USA; 3 Synthetic Biology Group, MIT Synthetic Biology Center; The Center for Microbiome Informatics and Therapeutics; Research Laboratory of Electronics, Department of Biological Engineering, and Department of Electrical Engineering and Computer Science, Massachusetts Institute of Technology, Cambridge, MA USA; 4 Broad Institute of MIT and Harvard, Cambridge, MA USA

**Keywords:** Biological techniques, Biotechnology, Cell biology, Computational biology and bioinformatics, Drug discovery, Immunology, Microbiology, Molecular biology, Diseases, Mathematics and computing

## Abstract

Venezuelan equine encephalitis virus (VEEV), a New World alphavirus of the Togaviridae family of viruses causes periodic outbreaks of disease in humans and equines. Disease following VEEV infection manifests as a febrile illness with flu-like symptoms, which can progress to encephalitis and cause permanent neurological sequelae in a small number of cases. VEEV is classified as a category B select agent due to ease of aerosolization and high retention of infectivity in the aerosol form. Currently, there are no FDA-approved vaccines or therapeutics available to combat VEEV infection. VEEV infection in vivo is characterized by extensive systemic inflammation that can exacerbate infection by potentially increasing the susceptibility of off-site cells to infection and dissemination of the virus. Hence, a therapeutic targeting both the infection and associated inflammation represents an unmet need. We have previously demonstrated that host defense peptides (HDPs), short peptides that are key components of the innate immune response, exhibit antiviral activity against a multitude of viruses including VEEV. In this study, we designed synthetic peptides derived from indolicidin, a naturally occurring HDP, and tested their efficacy against VEEV. Two candidate synthetic peptides inhibited VEEV replication by approximately 1000-fold and decreased the expression of inflammatory mediators such as IL1α, IL1β, IFNγ, and TNFα at both the gene and protein expression levels. Furthermore, an increase in expression levels of genes involved in chemotaxis of leukocytes and anti-inflammatory genes such as IL1RN was also observed. Overall, we conclude that our synthetic peptides inhibit VEEV replication and the inflammatory burden associated with VEEV infection.

## Introduction

Venezuelan equine encephalitis virus (VEEV) is an enveloped, positive-sense, single-stranded RNA virus with a genome of approximately 11.4 kb in length^1^. VEEV infection in vivo results in a biphasic disease; an initial phase with viral replication in peripheral organs, particularly lymphoid tissue, and an encephalitic phase with entry and replication in the central nervous system (CNS)^2^. Previously weaponized, VEEV is highly amenable for use in biological warfare. Furthermore, aerosol exposure has previously led to laboratory-acquired infections^3,4^. Currently there are no FDA-approved vaccines or therapeutics to combat VEEV infections. Two investigational vaccine candidates, TC-83 and C-84, are only given to at risk personnel. Whereas immunization with TC-83 elicits a strong host immune response, it exhibits high reactogenicity and thus is not approved for general use, whereas immunization with C-84 elicits only a weak immune response^5^.

VEEV infections induce the production of pro-inflammatory cytokines and thus are characterized by extensive inflammation. While inflammation is a host-protective mechanism by the immune system, the excessive inflammatory load elicited by VEEV infections can play a detrimental role in disease pathogenesis by a number of ways including: inflammation-induced tissue damage, disruption of blood–brain barrier (BBB) integrity, and aid in viral dissemination. During neurotropic phase of VEEV infection, an upregulation of pro-inflammatory genes in the CNS result in development of permanent neuronal damage and death^2,6^. Inflammatory mediators in the CNS also lead to disruption of the BBB. For example, in mice, disruption of BBB is evident in inflamed microvessels, highlighting the detrimental role inflammation plays in BBB integrity^2^. Studies looking at BBB integrity during VEEV infections demonstrated that infiltration of lymphocytes and inflammation coincides with BBB disruption^7^. Cellular adhesion molecules such as ICAM-1, which play a role in inflammation by mediating migration of lymphocytes into the brain, demonstrated an increase in expression in mice following VEEV infection^2^. Coincidently, mice deficient in ICAM-1 demonstrated a reduction in encephalitis and a less leaky barrier as compared to wild-type mice. This change in pathogenesis is marked with a decrease in inflammatory cytokines and an increase in anti-inflammatory genes^2^. Hence, a desirable therapeutic strategy to treat encephalitic infections following VEEV exposure should therefore be able to inhibit both viral multiplication and control infection-induced extensive inflammatory outcomes.

Host Defense Peptides (HDPs) are key components of the innate immune response and exhibit antimicrobial activity, and in some cases immunomodulatory activities, in particular, by upregulating signaling cascades that decrease inflammatory events^8^. Cathelicidins, a class of naturally-occurring HDPs, have demonstrated broad spectrum antimicrobial activity^8,9^. Expressed in a variety of tissues and cell types, cathelicidins are involved in the initial immune response to microbial infections. LL-37, one of the members of the human cathelicidin family, is a short amphipathic HDP that is naturally expressed during injury and infection^10^. Recently, LL-37 has been tested as an antiviral agent against both enveloped and non-enveloped viruses including: influenza type A virus (IVA), human immunodeficiency virus (HIV), Zika virus (ZIKV), dengue type-2 virus (DENV-2), hepatitis C virus (HCV), and vaccinia virus^11–16^. The antiviral activity of LL-37 has been attributed to its direct interaction with virions, which disrupts viral membranes, as well as its role in modifying and activating immune responses through the recruitment of immune cells such as monocytes and macrophages, downregulation of pro-inflammatory cytokines and upregulation of genes involved in anti-inflammatory responses^8,17^. LL-37 treatment leads to decreased production of pro-inflammatory cytokines such as tumor necrosis factor alpha (TNFα), interleukin 1 beta (IL1β), and interleukin 6 (IL6) in LPS-induced human monocyte cells in a dose-dependent manner^18^. In addition, treatment with synthetic LL-37 derivatives reduced expression of LPS-induced TNFα, and increased the expression of an anti-inflammatory cytokine, interleukin 10 (IL10)^18^. We have recently demonstrated the antiviral activity of LL-37 against VEEV in vitro using both the TC-83 vaccine strain and the wild-type Trinidad Donkey (TrD) strain of VEEV, where LL-37 treatment inhibited viral titers by approximately 100-fold and 1,000-fold respectively^19^. LL-37 inhibited VEEV replication in a cell-type-independent manner and modulated the type I interferon response by upregulating IFNβ1 expression. To further study the antiviral activity of HDPs against VEEV infection, we screened synthetic derivatives of indolicidin (ILPWKWPWWPWRR-NH2), a 13 amino acid residues long cathelicidin from bovine neutrophils, for antiviral activity against VEEV. This HDP has been reported as active against Gram-negative and Gram-positive bacteria, fungi, protozoa, and the human immunodeficiency virus (HIV-1)^20–23^.

In this study, we demonstrate the efficacy of synthetic peptides against VEEV infection in vitro. Our lead peptides inhibited viral replication in a number of cell lines, the attachment of VEEV virions to cells, and the inflammatory response resulting from VEEV infections. The inhibitory effect is demonstrated at the gene expression level by a reduction in the expression of pro-inflammatory genes and the increase fold-regulation of anti-inflammatory genes and genes involved in chemotaxis of leukocytes. The peptides also inhibited production of cytokines at the protein expression level, particularly those known to be induced during VEEV infections. The peptides demonstrate a novel therapeutic approach to limit VEEV replication and the associated inflammatory response.

## Results

### Synthetic peptides demonstrated anti-VEEV activity

The peptides used in this study were generated using a pattern recognition algorithm (PRA). PRAs are tools used to discover bioactive patterns by comparing molecules with certain biological function with other known bioactive molecules from databases or a desired template. Here, a library of twenty two synthetic peptides was generated based on the sequence similarities with the antimicrobial, antifungal and antiviral peptide indolicidin. The amino acid sequence of peptides from our library of antimicrobial peptides was submitted to Pratt 2.1, split according to their subgroups^24^. The flexibility parameters (flexible spacers and max flexibility) were set as two. The maximum number of wildcards was set at 5 and the pattern refinement was turned on. The remaining parameters were maintained as the default. To verify whether the pattern is specific, it was compared with the complete peptide library, using a PERL script. Patterns with correspondences to peptides less effective were considered non-specific. Initially, we conducted an analysis of the amino acid frequency for selecting a restricted group of amino acids for filling the wild cards (‘x’) in the patterns. The frequencies were extracted from (i) the complete antimicrobial peptide library; (ii) the peptides with antimicrobial inhibition equals or higher than 70%; and (iii) the peptides with antimicrobial inhibition equals or lesser than 30%. The amino acids whose frequencies in peptides from (ii) were higher than their frequencies from (i) and from (iii) were redistributed, and after that amino acids with frequencies equal or higher than 10% in the wild cards’ positions were selected for completing the patterns.

**Figure 1:**
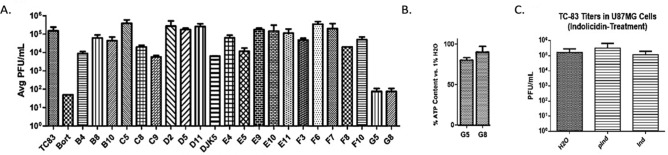
Indolicidin and first generation library efficacy. (**A**) Screen of first generation library in Vero cells generated using indolicidin as a template. Cells were pre-treated with the specified peptides and infected with TC-83 at an MOI of 0.1 as described in Materials and Methods. Data reported as average plaque forming units (Avg PFU/mL) of 3 replicates. Out of the peptides tested, G5 and G8 demonstrated TC-83 inhibition comparable to the positive control bortezomib. (**B**) Cytotoxicity of G5 and G8. (**C**) Efficacy of indolicidin against TC-83. PInd = cells pretreated with indolicidin; Ind = cells without pretreatment of indolicidin; n = 3.

**Figure 2:**
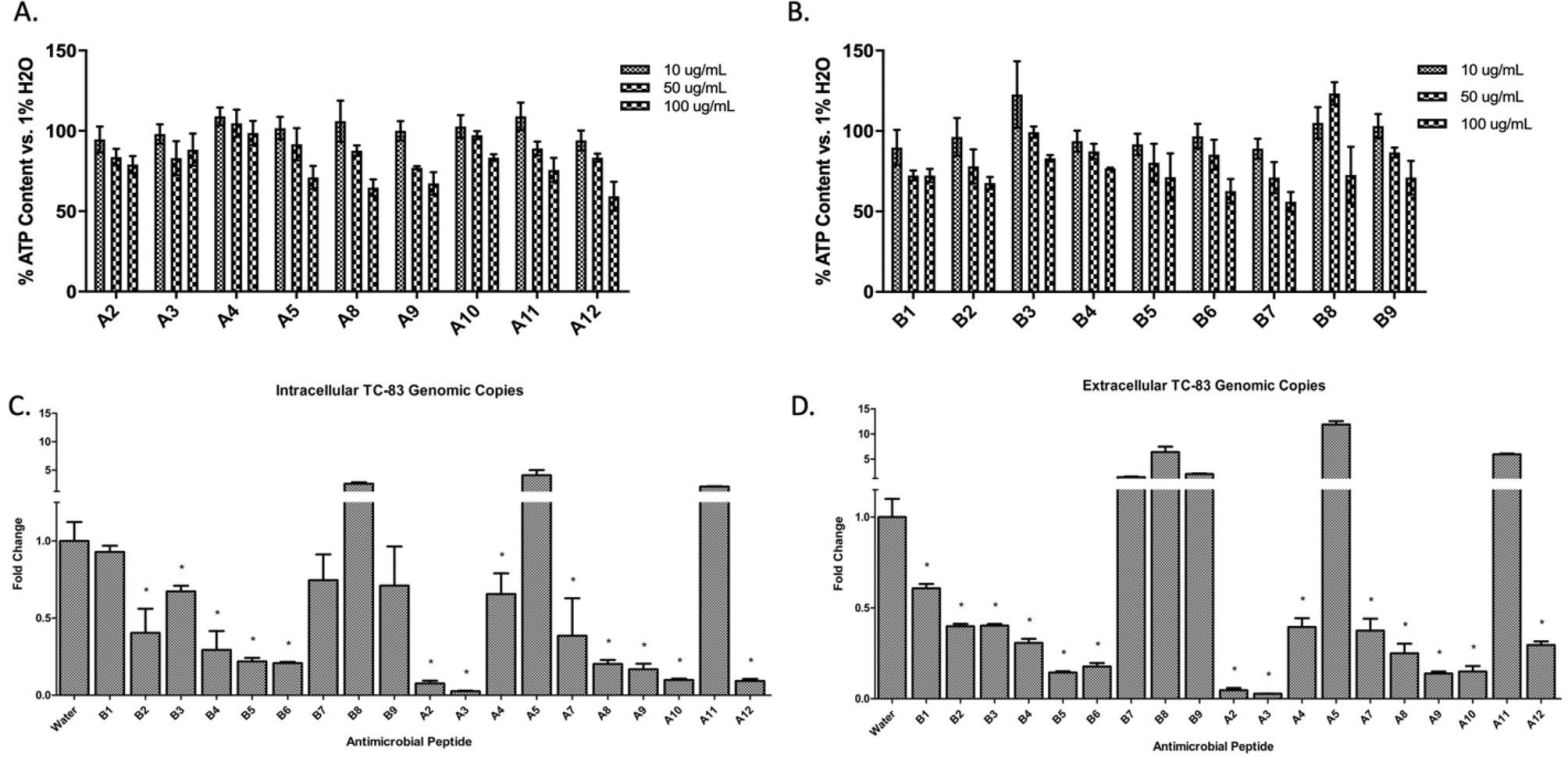
Efficacy of synthetic HDP library against VEEV TC-83. (**A**) and (**B**) Cytotoxicity of HDPs in BV2 cells. Values are vs. 1% water control in complete DMEM at 24hpi. Viability was measured at 24 h post-treatment. (**C**) Fold change in intracellular TC-83 genomic RNA levels in HDP-treated BV2 cells. (**D**) Fold change in extracellular TC-83 genomic RNA levels in HDP-treated BV2 cells. Fold change was measured in comparison to water control. n = 3; *p > 0.05.

The screening of the first library of antimicrobial peptides derived from indolicidin, resulted in two hits (G5 and G8) with VEEV TC-83 titer inhibition comparable to a known inhibitor, bortezomib (Fig. 1A). As G8 exhibited lower cytotoxicity (Fig. 1B), its sequence was utilized to generate a second generation of peptides, a VEEV-selective peptide library. The library consisted of 18 peptides: A1-A6, A9-A12, and B1-9. Additionally, the parental peptide indolicidin exhibited no antiviral activity against VEEV (Fig. 1C). The cytotoxicity of the second library of peptides was assessed at three different concentrations (10, 50, and 100 gμ/mL) against BV2 cells at 24 h post treatment, for which all peptides demonstrated > 90% cell viability at a concentration of 10 gμ/mL when compared to a control with cells in water (Fig. 2A,B). Next, we determined the efficacy of the synthetic peptides in controlling VEEV replication. BV2 cells were pre-treated with peptides at the non-toxic concentration (10 gμ/mL) for 2 h (hours) and subsequently infected with VEEV TC-83 (MOI = 0.1). At 16 h post infection (hpi) RT-PCR was used to measure levels of viral replication in infected cells. Six peptide candidates (A2, A3, A9, A10, B5, and B6) demonstrating more than 50% decrease in both intracellular and extracellular genomic TC-83 RNA copy numbers (Fig. 2C,D) were selected for further experiments in human microglia.

**Figure 3:**
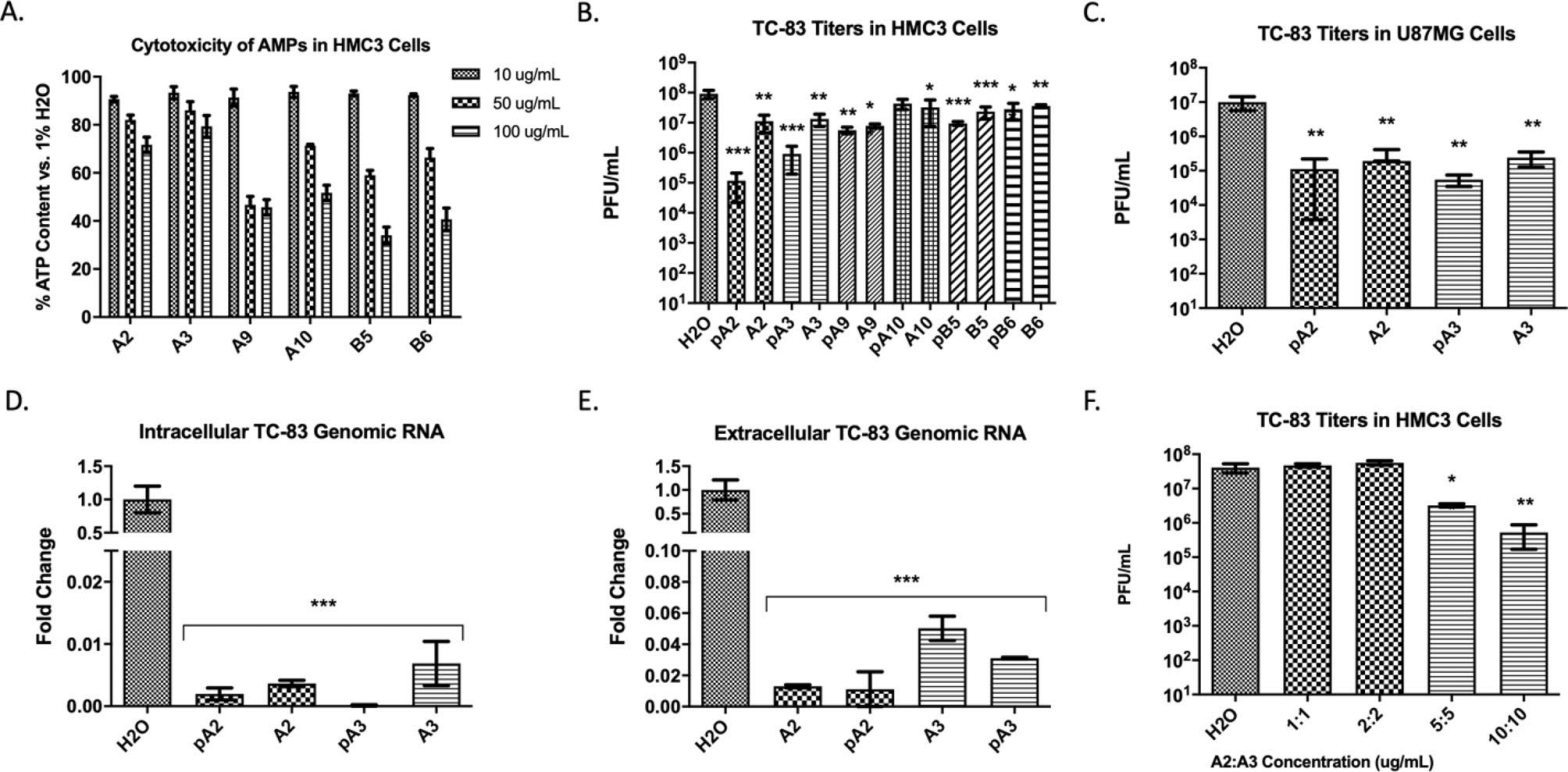
Efficacy of prioritized candidate peptides against VEEV TC-83. (**A**) Cytotoxicity of prioritized peptides in HMC3 cells; cells were pre-treated with peptides and toxicity was quantified at 24 h post-treatment; reported as % vs. water control. (**B**) TC-83 replication in peptide-treated HMC3 cells; reported as plaque forming units (PFU) per mL. Cells were treated with 10 µg/mL of each peptide. (**C**) TC-83 replication in U87MG cells treated with peptides A2 or A3. (**D**) and (**E**) Fold change in intracellular and extracellular genomic TC-83 RNA in peptide-treated HMC3 cells respectively; reported as fold change compared to water control. (**F**) Synergistic effects of A2 and A3 on TC-83 replication in HMC3 cells; reported as PFU/mL. n = 3; **p > 0.01; ***p > 0.001.

The cytotoxicity of the lead peptides was quantified in HMC3 cells as a representative neurovascular cell line for encephalitic VEEV infections (Fig. 3A). Cells were pre-treated (example: pA2) with the peptides for 2 h prior to infection or treated with peptides (10 gμ/mL) immediately before infection. Cells were subsequently infected with TC-83 (MOI = 0.1) for 1 h, and viral titers were measured at 16 hpi using a plaque assay. Treatment with two candidate peptides, A2 or A3, demonstrated a significant decrease (~ 1,000-fold for A2 and ~ 100-fold for A3, p > 0.001) in TC-83 infectious titers when compared to the water control (Fig. 3B). The antiviral activity of A2 and A3 was further tested in U87MG cells as VEEV replicates in astrocytes during neurotropic phases of infection (Fig. 3C). Similarly to BV2 cells, genomic TC-83 RNA copy numbers were significantly (p > 0.001) reduced in HMC3 cells upon A2 or A3 treatments (Fig. 3D,E). We further assessed the combined effects of these peptides and we observed that the individual inhibitory profiles were high than the combined effect (Fig. 3F). At 10 gμ/mL, co-treatment with A2 and A3 resulted in ~ 60-fold decrease of TC-83 titers, while A2 or A3 alone exhibited a 100-fold decrease when compared to the water control. These results indicate that both peptides, A2 and A3 can inhibit VEEV replication in multiple CNS cell lines. Additionally, the CC50 and IC50 of both peptides were determined for A2 and for A3 (Supplementary Fig. 1).

### The antiviral activity of two lead peptides A2 and A3 is alphavirus-specific and efficacious against the wild-type VEEV TrD strain

**Figure 4:**
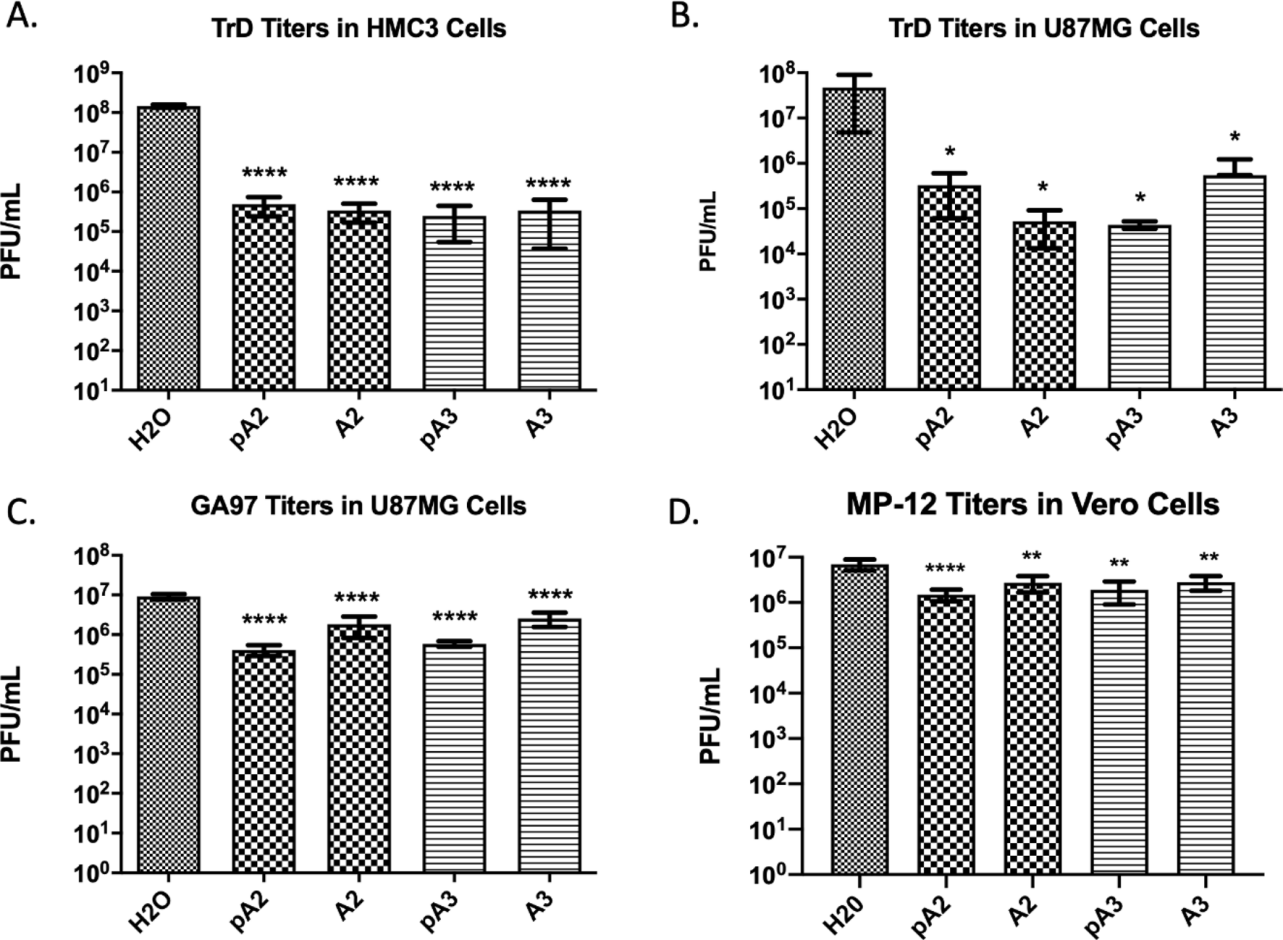
A2 and A3-mediated inhibition is alphavirus-specific and cell-type-independent. (**A**) and (**B**) Infectious titers of the VEEV TrD strain in HMC3 and U87MG cells respectively, reported as PFU/mL. Cells were pre-treated with peptides (pA2/pA3) for 2 h prior to infection; or cells were treated with peptides and immediately co-infected with virus. (**C**) EEEV (GA97 strain) titers in U87MG cells following treatment with peptides A2 and A3. (**D**). RVFV titers (MP-12 strain) in Vero cells following treatment with peptides A2 and A3. All treatments were performed at a concentration of 10 µg/mL of each peptide and infected with respective virus at MOI = 0.1. n = 3; *p > 0.05; **p > 0.01; ***p > 0.001; ****p > 0.0001.

We tested the antiviral activity of peptides A2 and A3 against the wild-type TrD strain of VEE, MOI = 0.1. Both peptides decreased TrD titers about 100-fold as compared to water controls in both HMC3 (p > 0.0001) cells and U87MG cells (p > 0.05) in the nontoxic range (10ug/ml), demonstrating that the antiviral activity of these peptides is cell-type-independent (Fig. 4A,B). In HMC3 cells the degree of inhibition was equal in pre-treated cells (pA2 or pA3) and cells that were treated immediately prior to infection. However, in U87MG cells, cells that were treated with A2 demonstrated TrD titer inhibition by a factor of tenfold higher than cells pre-treated with A2 before infection. In contrast, A3 pre-treatment had an inhibitory response tenfold higher than cells with no pre-treatment; the change however, was not significant. Inhibtition of TrD was also evident at a higher dose, MOI = 1 (Supplementary Fig. 2). To test if the antiviral activity was specific towards alphaviruses, these peptides were tested against the closely related new world alphavirus, eastern equine encephalitis virus (EEEV) and against a Bunyavirus, Rift Valley fever virus (RVFV). U87MG cells were treated with A2 and A3 and infected with EEEV. Treatment with either A2 or A3 inhibited the replication of EEEV by a factor higher than tenfold (p > 0.0001) (Fig. 4C). Cells pre-treated with either peptide exhibited lower (~ 4.5-fold) levels of EEEV replication. In contrast, treatment with A2 or A3 inhibited the replication of RVFV; however, the level of inhibition, less than tenfold (p > 0.01), was not comparable to that observed against alphaviruses (100-fold) (Fig. 4D). Thus, these results suggest that the antiviral activity of A2 and A3 could be selective to alphaviruses, however, more experiments are required for confirmation.

### A2 and A3 inhibit VEEV replication at an early stage during infection

**Figure 5:**
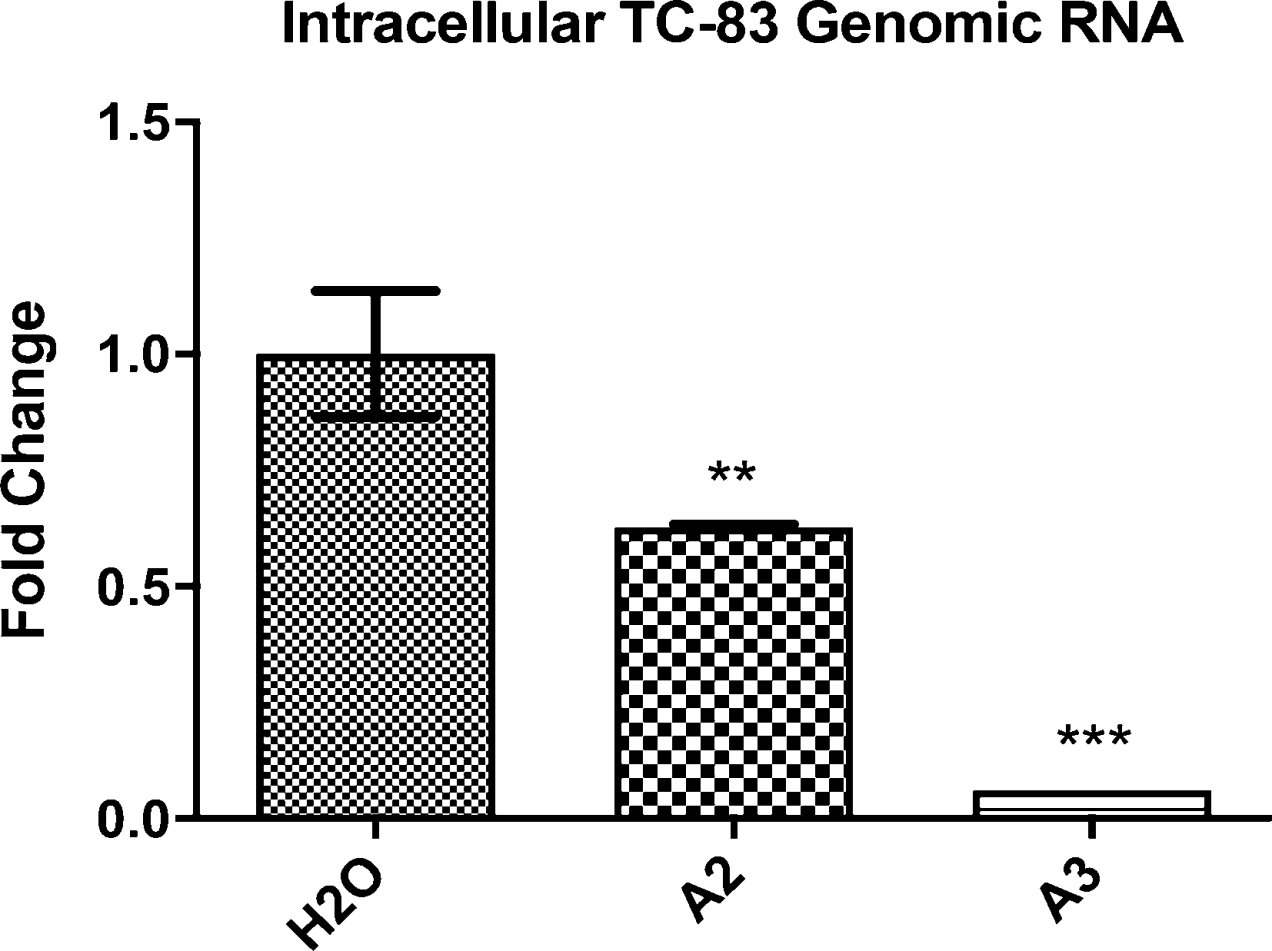
A2 and A3 inhibit VEEV particle attachment onto cells. Fold change in intracellular genomic TC-83 RNA copy number in U87MG cells, compared to water control. U87MG cells were treated with peptide (10 gμ/mL) and co-infected with VEEV TC-83 (MOI = 5) on ice. Cells were washed with PBS after infection and treated with media for 2 h before lysis, RNA extraction and RT-PCR. n = 3; **p > 0.01; ***p > 0.001.

We have previously demonstrated that LL-37 anti-VEEV activity was partly mediated through direct interaction with virions which decreased the number of particles entering cells during infection^19^. To assess whether the synthetic derivative peptides function in a similar manner, we performed an assay assessing viral attachment to cells in the presence of peptides. Cells were co-treated with the peptides A2 or A3 and virus, to allow for virus-peptide interactions and incubated at 4 °C for 1 h. This step was performed with ice-cold reagents and at 4 °C to ensure viral attachment but no viral internalization. Cells were subsequently washed to remove unattached virions and were incubated at 37 °C for 2 h to induce viral internalization. Cells were lysed and levels of genomic TC-83 RNA were quantified. Both peptide candidates, A2 and A3, inhibited VEEV attachment to cells, with A3 exhibiting a tenfold (p > 0.001) inhibition in the amount of viral RNA entering cells, a more robust and significant inhibition than A2 (p > 0.01) (Fig. 5). These data indicate that peptide-mediated inhibition of VEEV likely occurs at the attachment step during infection.

### Treatment with A2 or A3 inhibits upregulation of pro-inflammatory genes

**Figure 6:**
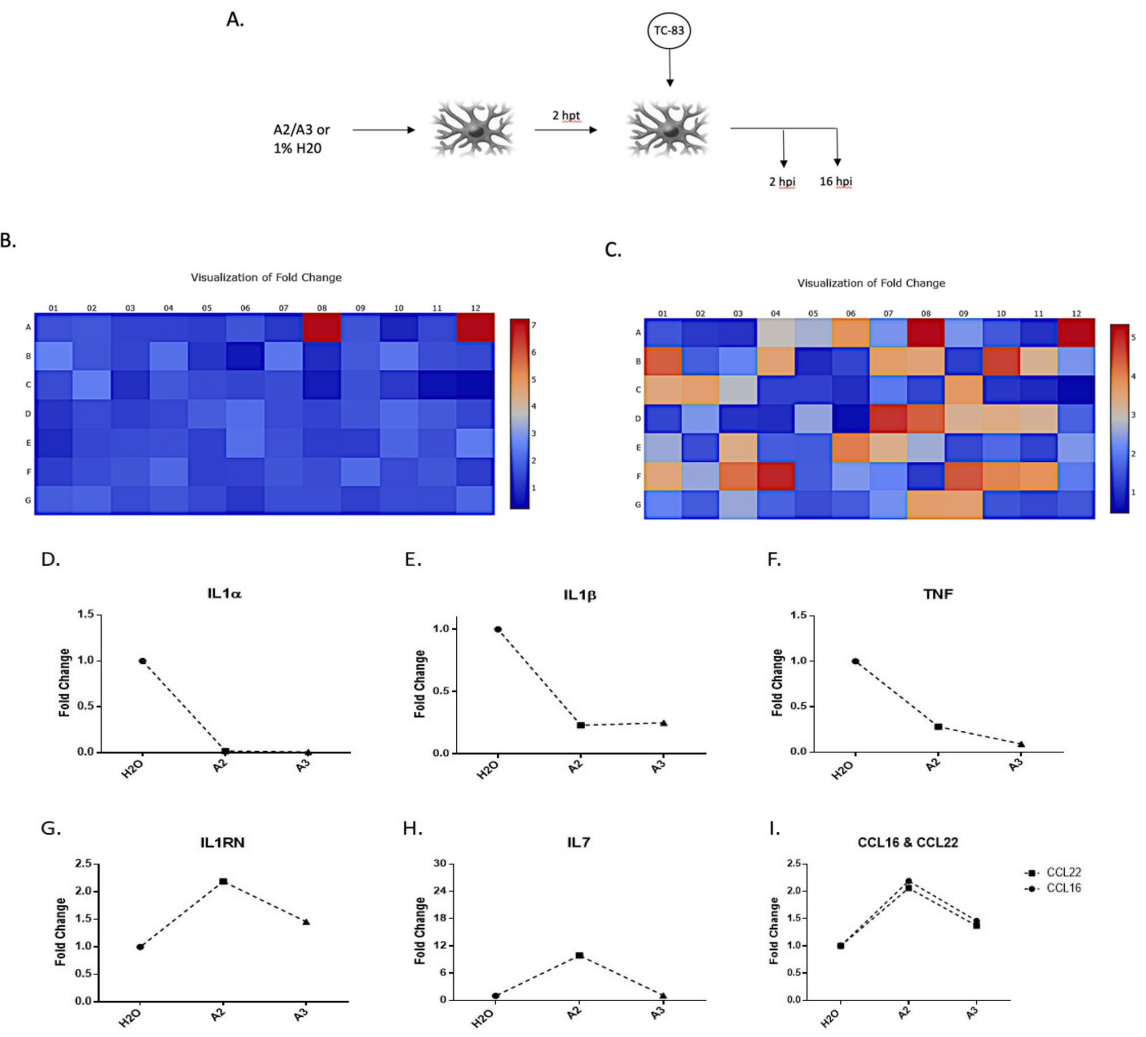
A2 and A3 treatment inhibits upregulation of the inflammatory response genes following VEEV infection. (**A**) Schematic of peptide treatment and TC-83 infection in U87MG cells (hpt = hours post treatment; n = 1). (**B**) Heat maps depicting the fold change in gene expression of PCR array performed following A2 pre-treatment of infected cells compared to water control. Red is maximum increase for IL7 gene expression. (**C**) Heat map of gene expression in A3 treated samples. Red depicts maximum expression for BMP2 gene; Orange depicts CCL16, CCL22, and IL1RN genes; Beige depicts IL7 and VEGFA genes. Scale: Blue, decrease in gene expression; Red, increase in gene expression. (**D**)–(**F**) Decrease in the gene expression of pro-inflammatory cytokines IL1α, IL1β, and TNF respectively in peptide treated cells. (**G**) and (**H**) Increase in the expression of anti-inflammatory genes IL1RN and IL7 respectively in peptide treated cells. (**I**) Increase in gene expression of genes involved in chemotaxis of leukocytes, CCL22 and CCL16, as a result of peptide treatment.

VEEV infections in vivo are characterized by extensive inflammation including systemic upregulation of pro-inflammatory cytokines^2,6^. High levels of inflammation following VEEV infection have been linked to increased viral dissemination, tissue damage, and disruption of BBB integrity^2,6,7,25^. Thus, an ideal anti-VEEV inhibitor should be able to inhibit viral replication as well as the associated extensive inflammatory burden. Interestingly, LL-37 exhibits a number of immunomodulatory properties including inhibition of inflammatory mediator expression, a characteristic observed when used as a treatment against a variety of pathogens^10,26^. To assess the anti-inflammatory activity of A2/A3, we used a qPCR-based gene array that targeted 84 inflammatory cytokines and receptor genes (see Supplementary Fig. 3 for plate scheme). Cells were pre-treated with each peptide (10 gμ/mL) prior to infection with TC-83 (MOI = 0.1). At 16 hpi, cells were lysed, RNA was extracted, converted to cDNA and expression levels were quantified using the array platform (Fig. 6A). A large number of genes were either up- or down-regulated by both A2 and A3. Of note, anti-inflammatory genes were up-regulated whereas genes that are involved in pro-inflammatory responses were down-regulated (Fig. 6B,C). In A2 treated cells, 71 out of the 84 genes exhibited greater than a twofold change in regulation (4 were upregulated and 67 were down regulated); whereas in A3 treated cells, a total of 60 genes demonstrated a twofold decrease in regulation (Supplementary Table 1). Noteworthy pro-inflammatory genes include: tumor necrosis factor (TNF), interleukin-1 alpha (IL1α), interleukin-1 beta (IL1β); chemokine ligand 4 (CCL4) also known as macrophage inflammatory protein 1-β; IL1 receptor antagonist (IL1RN); interleukin-17 (IL17); and bone morphogenetic protein 2 (BMP2), which all demonstrated a decrease in expression upon peptide treatment (Fig. 6D–F, Supplementary Table 1). Anti-inflammatory genes such as IL1 receptor antagonist (ILRN) and IL7 exhibited an increase in gene expression levels as a result of A2 and A3 treatments (Fig. 6G,H, Supplementary Table 1). Additionally, genes involved in chemotaxis such as CCL16 and CCL22 were upregulated as a result of A2 or A3 treatment compared to an untreated, infected control (Fig. 6I). These data suggest that both A2 and A3 exhibit anti-inflammatory properties, as evident by the decrease in the expression of genes involved in upregulation of inflammation, and an increase in expression of anti-inflammatory genes.

**Figure 7:**
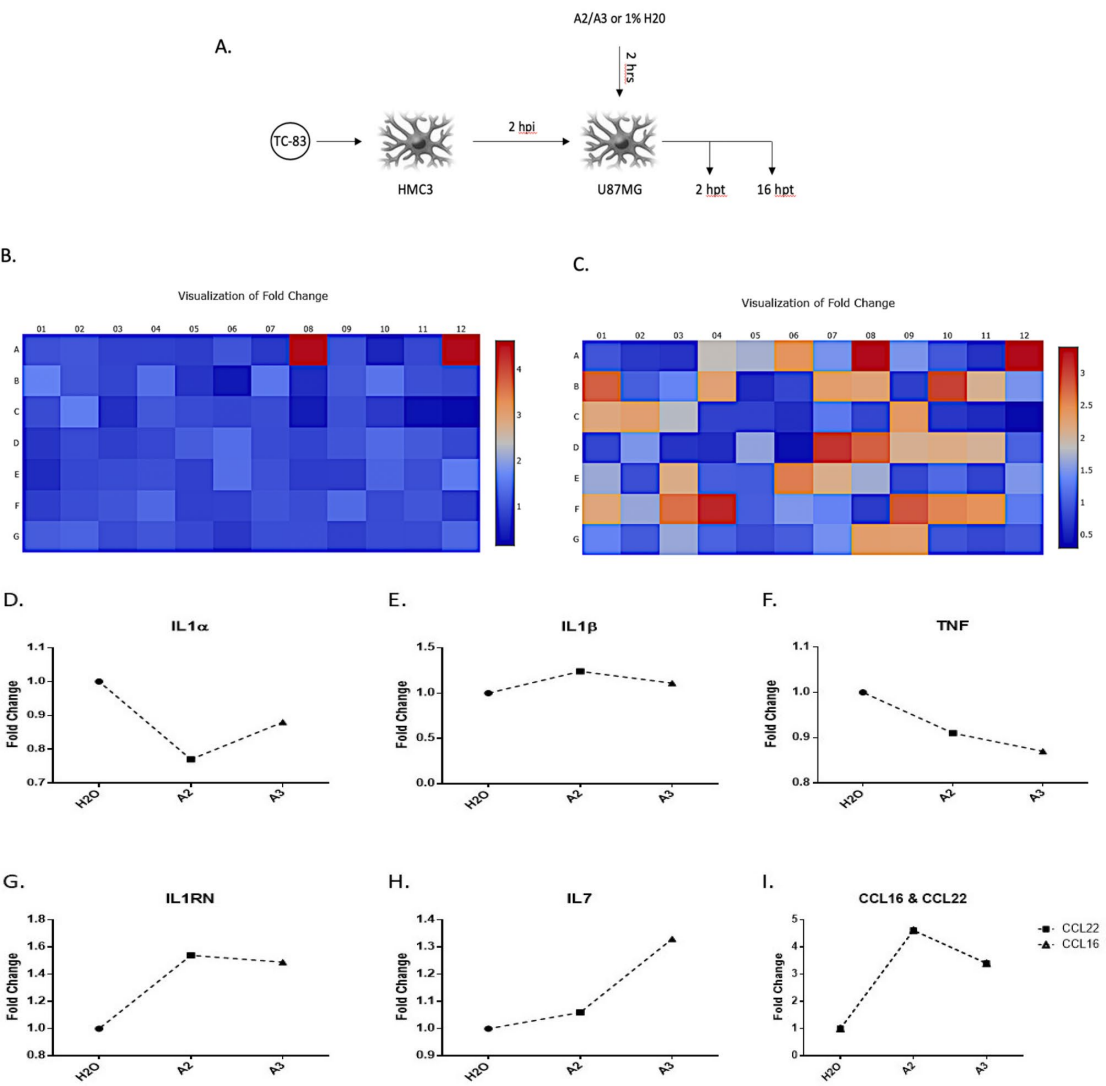
A2 and A3 treatment inhibits upregulation of inflammatory response genes in uninfected cells. (**A**) Schematic of peptide treatment in U87MG cells that are overlaid with supernatants from infected HMC3 cells to assess inflammation from bystander, peptide-treated cells (htp = hours post treatment; n = 1). (**B**) Fold change in PCR array gene expression in A2-treated sample as depicted by a heat map. Red (A7 and A12) represents expression of CCL16 and CCL22 genes. (**C**) Heat map of gene expression in A3-treated samples. Gene changes in array as follows; Red: CCL16, CCL22; Coral: CCR2, CXCL9; Orange: CCL23, CXCR1, IL3, IL9; Peach: CCL13, CCL3, CCL7, CCL8, CX3CR1, IL17A, IL9R, LTA; Beige = CCR3, CCR5, CCR6, CXCR2, FASLG, IFNA2, IL13, IL17C, IL21, TNFSF11, TNFSF13. Scale = Blue, decrease in gene expression; Red, increase in gene expression. (**D**)–(**F**) Decrease in expression levels of pro-inflammatory cytokines IL1α, IL1β, and TNF respectively in peptide treated cells. (**G**) and (**H**) Increase in the expression of anti-inflammatory genes IL1RN and IL7 respectively in peptide treated cells. (**I**) Increase in expression of genes involved in chemotaxis of leukocytes, CCL22 and CCL16, as a result of peptide treatment.

Furthermore, inflammatory cytokines produced by infected cells can act on a number of cells including uninfected, bystander cells causing those cells to secrete inflammatory mediators^27^. We have previously demonstrated that the VEEV-induced neuro-inflammatory load is a cumulative result of two types of inflammation; direct inflammation, mediators released by infected cells; and indirect inflammation, cytokines released from bystander cells which are primed by infection-induced, circulating cytokines^28^. Resident microglial cells secrete inflammatory cytokines that act on naïve, bystander astrocytes causing these cells to further express inflammatory mediators thus contributing to the overall inflammatory outcome^28^. Hence to attest whether A2/A3-mediated inhibition of inflammatory mediators is not a decrease resulting of viral replication inhibition, and to assess the effect of A2 and A3 on bystander-induced inflammatory load, we overlaid supernatants from infected microglial cells on peptide-treated U87MG cells and quantified gene expression using the above gene array (Fig. 7A) and extracted RNA at 16 hpt. Interestingly, while A2 changed the gene expression profile across the board (Supplementary Fig. 4; Fig. 7B), only eight genes were above/below a twofold regulation change (Supplementary Table 2). A3 on the other hand changed the expression profiles of 30 genes by a twofold factor (28 upregulated and 2 were down-regulated) (Supplementary Table 2; Fig. 7C). For both peptides, noteworthy pro-inflammatory genes whose expression was decreased include TNF, IL1α, and IL1β (Fig. 7D–F), whearas anti-inflammatory and chemotactic genes whose gene expression was increase include IL1RN, IL7, CCL16, and CCL22 (Fig. 7G–I). These results suggest that the peptides exhibit anti-inflammatory properties in addition to inhibiting VEEV infection.

### Pro-inflammatory cytokine production is decreased upon treatment with A2 or A3

**Figure 8:**
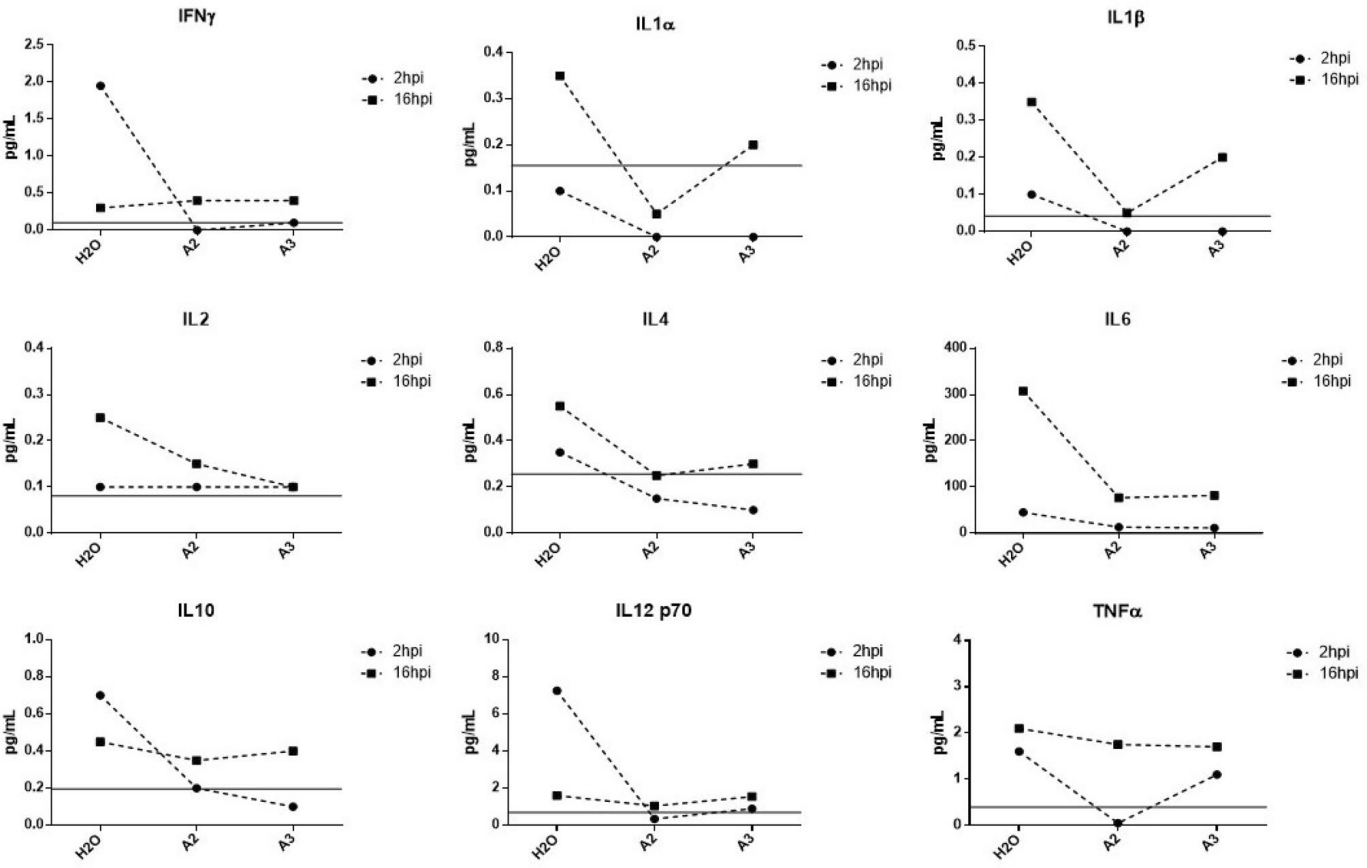
A2 and A3 inhibit secretion of cytokines from TC-83 infected cells. The concentration of IFNγ, IL1α, IL1β, Il2, IL4, IL6, IL10, IL12 p70, and TNFα in pg/mol in U87MG cells as a result of A2 and A3 treatment in comparison with untreated control. Limit of detection depicted by gray line; n = 1.

We demonstrated above that treatment with A2 or A3 inhibits upregulation of pro-inflammatory mediators at the gene expression level. Here we wanted to assess if this inhibition is quantifiable at the protein production level. To assess cytokine production, we employed a multiplex ELISA kit assaying 10 pro-inflammatory and anti-inflammatory cytokines known to be induced during VEEV infections^6^. As expected, cytokine production increased in TC-83 infected U87MG cells when compared to uninfected cells. However, this was only observed at 16 h post infection. During the early stages of infection, 2hpi, cytokine levels demonstrated little or no change upon treatment in infected cells compared to untreated cells (Fig. 8). At 16hpi, an increase in production of IL1α, IL1β, IFNγ, IL2, and IL12p10 was observed in infected cells which decreased upon peptide treatment. Interestingly, the production of anti-inflammatory cytokines, IL4, IL6, and IL10, was decreased in treated cells as compared to untreated cells (Fig. 8) indicating that the peptides decrease the production of cytokines without discrimination.

**Figure 9:**
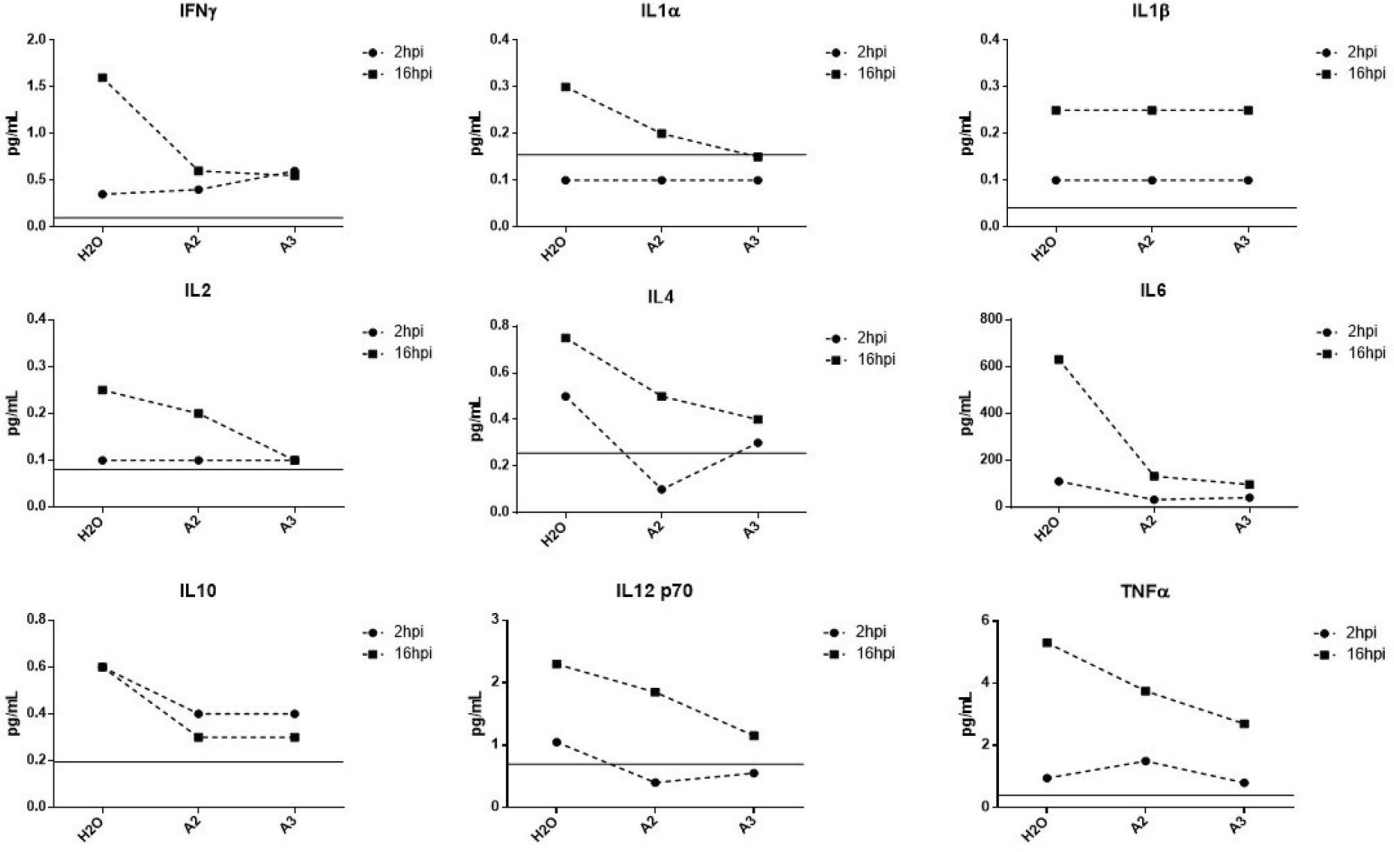
A2 and A3 treatment inhibits production of cytokines from uninfected cells. The protein expression levels of IFNγ, IL1α, IL1β, IL2, IL4, IL6, IL10, IL12 p10, and TNFα in U87MG cells that were overlaid with supernatants from HMC3 cells, reported as concentration in pg/mol compared to untreated control. Limit of detection depicted by gray line; n = 1.

To assess the production of inflammatory mediators from uninfected cells, we employed the indirect inflammatory assay as described above (for infection scheme, see Fig. 7A). The concentration of all cytokines tested decreased upon peptide-treatment as compared to untreated cells (Fig. 9). Although there was a decrease in the gene expression of IL1β, a change in protein expression was not evident. This is expected as the inflammatory burden from bystander cells has been demonstrated to be greater than that of infected cells, as we previously reported^28^. Similarly, A2 and A3 both decreased the production of cytokines, pro-inflammatory and anti-inflammatory, with A2 exhibiting a more robust inhibitory response. Hence, these synthetic peptides can inhibit VEEV-induced inflammation at the gene and protein expression levels.

## Discussion

Host defense peptides have recently gained attention as potent antiviral agents^8,22^. Cathelicidins have demonstrated antiviral activity against a number of viruses including Venezuelan equine encephalitis virus^19^. In addition to exhibiting potent antiviral properties, cathelicidins demonstrated immunomodulatory activity such as modulation of chemotaxis and the inflammatory response^9,10,26^. We have previously demonstrated the anti-VEEV activity of the cathelicidin LL-37 in which the peptide inhibits VEEV by directly interacting with viral membranes thereby preventing entry, a property seen with many viruses^8,19^. However, the broad nature of cathelicidins render them less ideal as a targeted therapeutic. New techniques in peptide design results in compounds with high activity and specificity which boosts the biological interest on their use as potential antimicrobials^29−34^. Hence, using PRA, a library of synthetic peptides, based on the sequence of indolicidin, was generated with specificity to VEEV and potentially other alphaviruses. Computational-based approaches to designing peptides can be used to generate synthetic versions that are more ideal than the natural templates used for design ^35,36^. Algorithm tools utilize biological and chemical properties that are known to influence activity of peptides such as sequence length, net charge, and hydrophobicity^35^. These factors along with other computational biology approaches and method-based strategies allow for the generation and selection of a large number of peptides designed with pathogen specificity. The synthetic library utilized in this study was designed using patterns of antimicrobial peptides and indolicidin as a template to target VEEV. The provided data ascertains the antiviral and anti-inflammatory properties of our synthetic peptides.

By screening a library of 18 synthetic peptides, we identified 6 peptides with potent antiviral activity. We narrowed our library to two peptides (A2 and A3) as they decreased both intracellular genomic TC-83 RNA copies as well as infectious VEEV particles (Fig. 3). The antiviral activities of A2 and A3 are sequence specific as scrambled sequences of both A2 and A3 had minimal effects on VEEV activity (Supplementary Fig. 5). This is expected as HDPs, including LL-37 in our previous studies, exhibit structure-based inhibitory properties where a specific sequence within a peptide is responsible for such activities. The peptides also inhibited the wild-type strain of VEEV, TrD, in multiple cell lines, indicating that the peptides can be utilized in a number of tissues. A2 and A3 also exhibited partial anti-EEEV and minimal anti-RVFV activities. It was expected that the peptides would demonstrate anti-EEEV activity due to the genomic similarities between the two new world alphaviruses. However, while both A2 and A3 demonstrated anti-EEEV activity, a more robust inhibitory response was evident with VEEV. This is possibly due to two factors: 1) the peptides were designed to target VEEV, and 2) the enhanced neurovirulence of EEEV compared to VEEV^37^. On the other hand, when we tested A2 and A3 against an unrelated virus, RVFV, the inhibitory response was further diminished than EEEV. Since all three viruses contain a host-derived membrane, the small inhibitory effect of the peptides on RVFV could be attributed to the direct interaction with viral membranes, as a primary mechanism of action of HDPs, cathelicidins included, is entry inhibition by means of membrane disruption. This mechanism of action, however, requires further validation. Thus, A2 and A3 exhibit selectivity towards VEEV inhibition.

Furthermore, VEEV infections are characterized by extensive inflammation which can be detrimental. However, not all inflammation is bad. In fact, inflammation is a vital, biological protective response whereby the immune system responds to stimuli such as infection and injury^38,39^. A transient or acute inflammatory response that is resolved once the stimuli is eliminated/controlled is hence required for normal biological immune response. However, excessive and unresolved inflammation can be detrimental and can contribute to the pathogenesis of diseases^38,40,41^. During VEEV neurotropic infections, excessive inflammation in the brain exacerbates the disease outcome by causing damage to neurons and contributes to morbidity^42^. Inflammatory chemokines and cytokines have indeed been implicated to contributing to encephalitis during VEEV infections^42^. While inflammation is beneficial to the host during infections, persistent or excessive inflammation can be damaging. Hence, the ideal anti-VEEV candidate should not only inhibit viral replication, but also limit the associated inflammatory response generated by infection. This criterion is met by our synthetic candidates.

Pro-inflammatory cytokines such as IL1α, IL1β, TNF, and IL17 demonstrated a decrease in expression upon treatment with both A2 and A3 (Figs. 6D–F; 7D–F). This was also reflected at the protein expression level where the production of cytokines such as IL1α, IL1β, TNFα, IFNγ was decreased upon peptide treatment compared to untreated infected controls (Fig. 8). This decrease was also mirrored in the assays assessing inflammatory response from bystander cells in absence of infection, thus indicating that the exhibited decrease in gene and protein expression is not just a result of a decrease in viral replication. Furthermore, expression of genes encoding for inflammatory chemokines such as CCL2, CCL3, CCL4, CCL5, CXCL10, and CCL11 were decreased in A2/A3-treated cells, particularly CCL3, CCL4, and CCL5 (Supplementary Tables 1 and 2). These pro-inflammatory chemokines have been reported to be upregulated in microglia during a number of neurotropic infections including West Nile virus (WNV) and the alphaviruses Semliki Forest virus (SFV) and Sindbis virus (SINV)^43,44^. An increase of the expression of these pro-inflammatory chemokines results in recruitment of leukocytes to sites of infection as well as an increase in inflammatory response. The reported decrease, hence, is beneficial in limiting inflammation in the context of VEEV infections and as a result possibly decrease neuronal damage and viral dissemination to tissue distal from infection sites. In addition, levels of CCL2, CCL4, and CXCL0 were reported to be significantly increased in Chikungunya (CHIKV) infected adults and infants as compared to uninfected individuals^45^. More importantly, the upregulation of CXCL9, CXCL10, CXCL11, CXCL13, CCL3, and CCL5 during VEEV infections has been reported to correspond with increased blood–brain barrier compromise^42^. The noted decrease in the expression of these cytokines upon peptide treatment hints at their application as therapeutics during VEEV infections, particularly at preventing a loss in BBB integrity. Based on our results, all of the above-mentioned chemokine expression levels were decreased upon A2 or A3 treatment compared to untreated cells, thus highlighting the anti-inflammatory properties of these peptides.

While a decrease in pro-inflammatory mediators was evident, anti-inflammatory cytokines such as the IL1 receptor antagonist (IL1RN) demonstrated a robust increase in gene expression (Figs. 6G and 7G). IL1RN is a potent anti-inflammatory mediator that modulates the activity of the major inflammatory cytokine IL1. IL1RN was increased by approximately 1.5-fold in bystander cells and by approximately twofold in infected cells, possibly playing a role in the decrease of IL1 production and thereby IL1-indced inflammation. Interestingly, a number of these cytokines and chemokines play contrasting roles during certain viral infections. For example, during Japanese encephalitis virus (JEV) infections, CCL2 has been detected in patients as well as mice deficient in CCL2 have demonstrated an increased susceptibility to JEV infections^46^. Additionally, the chemokines, CCL16 and CCL22, demonstrated a greater than twofold increase in gene expression upon peptide treatment. CCL16 and CCL22 act as chemoattractants to a number of leukocytes and have been implicated in pro- and anti-inflammatory events (Figs. 6I and 7I). HDPs, such as LL-37 and indolicidin, have complex immunomodulatory properties that may contribute to inflammation by means of recruiting leukocytes and decrease pro-inflammatory cytokine production^9^. As such, the contradicting modulatory activities of A2 and A3 could be attributed to these different properties. For example, att the protein expression level, both A2 and A3 decreased the expression of pro- and anti-inflammatory genes indiscriminately. The increase in the expression of chemotaxis genes upon peptide treatment could possibly explain the noted decrease in anti-inflammatory mediators, but further investigation is required to assess these different properties of HDPs. Nonetheless, it is important to note the evident decrease in pro-inflammatory mediators.

Based on the attachment assay data, A3 exhibits more robust antiviral properties compared to A2 (Fig. 5), while A2 demonstrated a more potent anti-inflammatory response. The difference in inhibitory properties can be attributed to the peptide sequences. Both A2 and A3 (Table 1) are 16 amino acids long and carry the same net charge (+ 4), however, A3 contains more hydrophobic residues thus maintain a higher hydrophobicity index and ratio than A2. As hydrophobicity is the main driving force in terms of propensity to interact with membranes of microbes, A3 is expected to exhibit more antiviral activity^47^. Nonetheless, the different secondary structures these peptides can adopt could also have an impact on their activities. Additionally, it is possible that A2/A3-mediated inhibition of viral replication potentially occurs at multiple steps during VEEV infection. These include post-attachment/entry steps such as fusion with endosomal membranes or during viral transcription and replication, not ruling out potential effects of the peptides on host processes that ultimately inhibit viral replication. In addition, the constant exposure of cells to the peptides further inhibits newly produced viruses from infecting cells.

**Table 1:** Sequences of indolicidin, G8, A2, and A3.

Peptide	Sequence	Length	Charge	Hydrophobicity Kcal/mol	Hydrophobic ratio (%)
Indolicidin	ILPWKWPWWPWRR	13	+ 3	1.92	54
G8	FQVVKFRFWVWWFRWR	16	+ 4	0.32	75
A2	FAVVKFRFWVWWFRWR	16	+ 4	0.05	75
A3	FQAVKFRFWVWWFRWR	16	+ 4	1.28	69

The sequences and properties of the original peptide, indolicidin, and the parent peptide (G8), of which the peptide candidates are derived from.

This study sheds light into the complexity of immune responses elicited by VEEV-infections and HDPs. Successful clearance of infections requires the activation of inflammatory leukocytes to some level, however, the excessive cytokine storm elicited by some viruses is detrimental to the host. The selective decrease/increase in inflammatory mediators by A2 and A3 adds to the complexity of immune inflammatory responses. For instance, an upregulation in some pro-inflammatory chemokines such as CCL16 and CCL22 can aid in viral clearance by serving as chemoattractants to immune cells, while a decrease in other chemokines could serve to limit excessive, detrimental inflammation. More experiments are required to further elucidate the role of A2 and A3 during VEEV-induced inflammatory responses. Future studies will aim to assess the effects of A2 and A3 treatment on inflammation at the BBB and maintenance of BBB integrity.

## Materials and methods

### Cell culture and reagents

Mouse brain microglia (BV2, EOC 20 CRL-2469), human microglial cells (HMC3, CRL-3304), human astrocytoma cells (U87MG, HTB-14), and African green monkey kidney cells (Vero cells, CCL-81) were obtained from American Type Culture Collection (ATCC). HMC3 cells were maintained in 1X Eagle’s Minimum Essential Medium (1X EMEM, Life Technologies, 670086) supplemented with heat-inactivated 10% fetal bovine serum (FBS, Gibco) and 1% penicillin/streptomycin (Corning, 30-002-CI). U87MG cells were grown in Dulbecco’s Modified Eagle’s Medium (DMEM, Quality Biological, 112-012-101) supplemented with 10% FBS, 1% penicillin/streptomycin, and 1% L-glutamine (Corning, 25-005-CI). Vero and BV2 cells were maintained DMEM supplemented with 5% FB Essence (VWR, 10799-390), 1% penicillin/streptomycin, and 1% L-glutamine. Cell cultures were grown at 37 °C and 5% CO_2_.

### Solid-phase peptides synthesis

Peptides were synthesized using fluoromethyloxycarbonyl (Fmoc) strategy in Rink Amide resin by Biopolymers (MIT). The purity of the peptides was higher than 95%.

### Viability assays

Toxicity of peptides was measured using Cell Titer-Glo Luminescent Cell Viability Assay (Promega, G7572) as per manufacturer’s instructions after 24 h treatment with peptides. Assay was performed as previously described^19^.

### Viruses

The VEEV TC-83, live attenuated strain, and the wild-type Trinidad Donkey strain were obtained from BEI Resources. The MP-12 strain of RVFV was rescued and titerd as previous described (Benedict et al. 2015, PMID: 26217133). All VEEV-TrD and EEEV (strain GA97) experiments were performed under BSL3 conditions.

### Viral infections and peptide treatments

Appropriate cells were seeded in multi-well plates and grown overnight to ~ 90–100% confluency prior to infection. For infections, virus stocks were diluted in appropriate cell culture medium to an MOI of 0.1, unless otherwise stated. Cells were infected for 1 h to allow for virus absorption after which cells were washed with 1 × PBS and fresh cell culture medium was added. Supernatants were collected at indicated time points and plaque assays were performed to measure viral growth as previously described^19^.

Peptide treatments were performed as follows: pre-treatment; and treatment at time of infection. During pre-treatment, cells are pre-treated with peptide (10 gμ/mL diluted in media, unless otherwise stated) or 1% water, for 2 h prior to infection (example: pA2). Infectious inoculum was added directly to the treated media to allow for virus-peptide interactions. In conditions where cells were treated and infected concurrently (example: A2), peptides were added to cells together with infectious inoculum. For all conditions, after infection, viral inocula were removed and cells were re-treated with the appropriate peptide/water until sample collection.

### Quantitative real-time PCR (qRT-PCR)

Extracellular and intracellular RNA was extracted using TRIzol reagent (Invitrogen, 15596026) and Zymo Direct-Zol RNA Miniprep kit (Zymo Research, R2050) per manufacturer’s instructions. Primers targeting VEEV capsid and PCR cycling conditions were used as previously described^19^. Data was quantified using a standard curve based on cycle threshold (Ct) numbers.

### Viral attachment assay

U87MG cells were seed at a density of 1E5 cells/mL in 12-well plates and grown overnight at 37 °C to 90–100% confluency. Cell monolayers were pre-chilled at 4 °C for 1 h. Cells were co-infected/treated with TC-83 (MOI = 5) and each peptide (10 gμ/mL) on ice for 1 h at 4 °C. Infectious media was removed and cells were washed twice with ice-cold PBS and culture medium was added to cells. Cells were incubated for 2 h at 37C. After 2 h, media was removed and discarded, cell were washed and lysed with TRIzol and intracellular genomic RNA copies were quantified as described above.

### Inflammatory gene array

The inflammatory array used in this study, RT^2^ Profiler PCR Array, targeted 84 human inflammatory cytokines and receptors (Qiagen, PAHS-011ZC, 330231). HMC3 cells were seed in 12-well plates at a density of 1E5 cells/mL. Cells were pre-treated with either peptide (10 gμ/mL) or 1% water for 2 h. Subsequently, cells were infected with TC-83 (MOI = 2) and incubated for 1 h at 37 °C. The viral inoculum was removed from cells and cells were re-treated with respective peptide or 1% water and incubated at 37 °C. Cells treated with lipopolysaccharide (LPS) were used as a positive control. At 16hpi, cells were lysed with TRIzol and RNA was extracted using Zymo Direct-Zol Miniprep kit. cDNA was synthesized using RT^2^ First Strand Kit (Qiagen, 330401). The PCR array was quantified as per manufacturer’s instructions using RT^2^ SYBR Green qPCR master mix (Qiagen, 330521) and StepOnePlus Real-Time PCR system.

### Multiplex ELISA

A multiplex ELISA, Ciraplex Human Cytokine 10-Plex, was used to quantify inflammatory cytokine production following TC-83 infection of untreated and peptide-treated cells (Aushon, 101-3FF-1-AB). The quantity of the following cytokines was measured: IFNγ, IL1α, IL1β, IL2, IL4, IL6, IL10, IL12p70, and TNFα. Cells were seeded in 96-well plates at a density of 1E4 cells/mL and pre-treated with respective peptides (10 gμ/mL) for 2 h prior to infection. Cells were infected as described above and inoculum was removed and replaced with peptide-treated media. An uninfected mock and an infected 1% water vehicle were included as negative and positive controls, respectively. Supernatants were collected at 2hpi and 16hpi and diluted 1:1 in sample diluent as per kit’s instructions. Samples and cytokine standards were added to pre-washed assay plates and placed on a shaker (515 rpm) for 2 h at room temperature. After incubation, a Biotinylated Antibody Reagent was added to each well. The plate was further incubated on the shaker for 30 min. A Streptavidin-HRP Reagent was added to each well and the plate was re-incubated for an additional 30 min. A 1:1 mixture of SuperSignal Substrate and Luminor Enhancer was added to the plate; the plate was read using a Cirascan Imaging System and the data was analyzed using Cirasoft Analysis Software. The plate was washed 4 × with a wash buffer after each incubation step.

### Statistical analysis

All graphs and analyses were generated using GraphPad Prism software version 7. Inflammatory array images and data were obtained using Qiagen Data Analysis Center. Replicate numbers (n) for each experiment are specified in the figure legends. Analyses were performed using an unpaired one-way ANOVA test. Data are represented as a mean with ± SD. Statistical significance is indicated as follows: *p < 0.05; **p < 0.01; ***p < 0.001; ****p < 0.0001.

## Supplementary information

Supplementary Information.
