# Supplementary material for: Synthetic Host Defense Peptides Inhibit Venezuelan Equine Encephalitis Virus Replication and the Associated Inflammatory Response
Source: Sci Rep. 2020 Dec 8;10:21491. doi: 10.1038/s41598-020-77990-3 (PMC7722873; doi:10.1038/s41598-020-77990-3)
Supplement: Supplementary file 1 — Supplementary Information. [file 41598_2020_77990_MOESM1_ESM.docx]

**Supplementary Figures:**


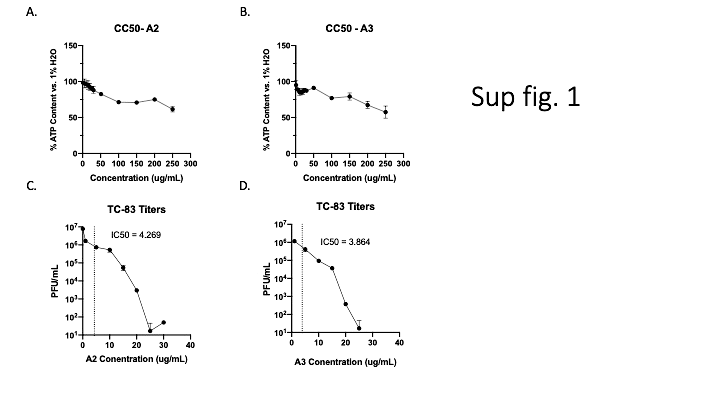


**Supplementary Figure 1:** **CC50 and IC50 of A2 and A3.** A) and B) Cytotoxicity of A2 and A3 in HMC3 cells with CC50’s greater than 250 μg/mL for both peptides. C) and D) Titer of TC-83 in HMC3 cells treated with A2 and A3 with CC50’s indicated in the respective figures. CC50 and IC50 calculated using nonlinear regression on GraphPad Prism; n=1.

**
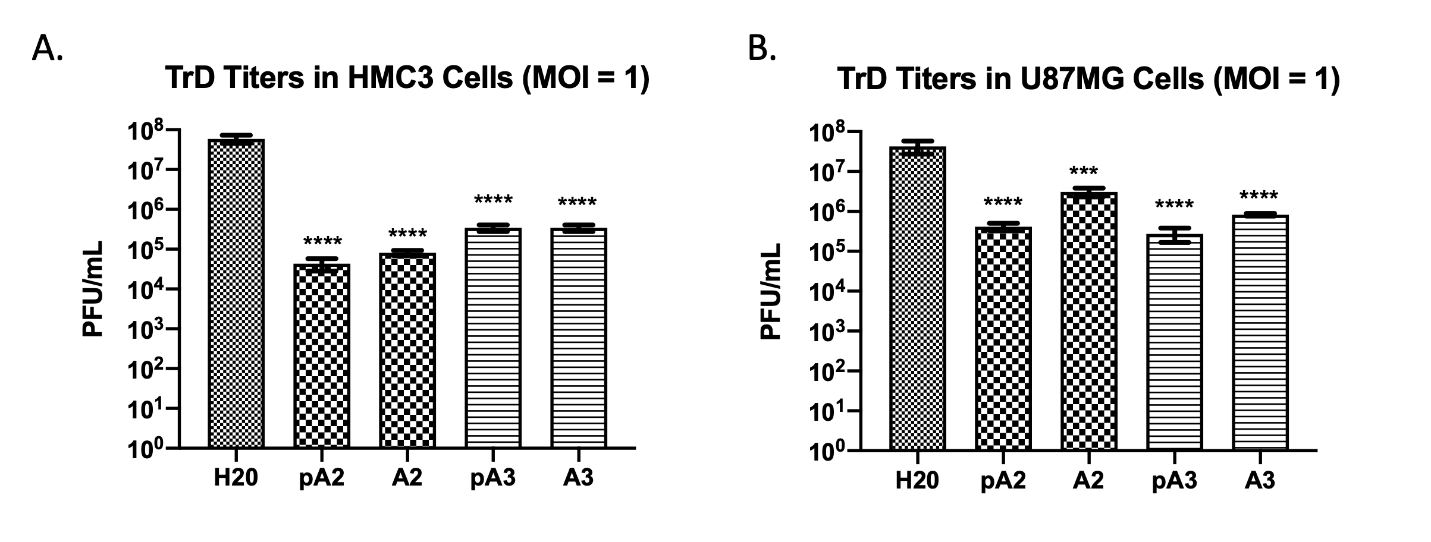
**

**Supplementary Figure 2. Titers of TrD at MOI = 1 in HMC3 and U87MG cells.** A) and B) TrD titers in HMC3 and U87MG cells at MOI of 1. n=3; ***p>0.001; ****p>0.000.


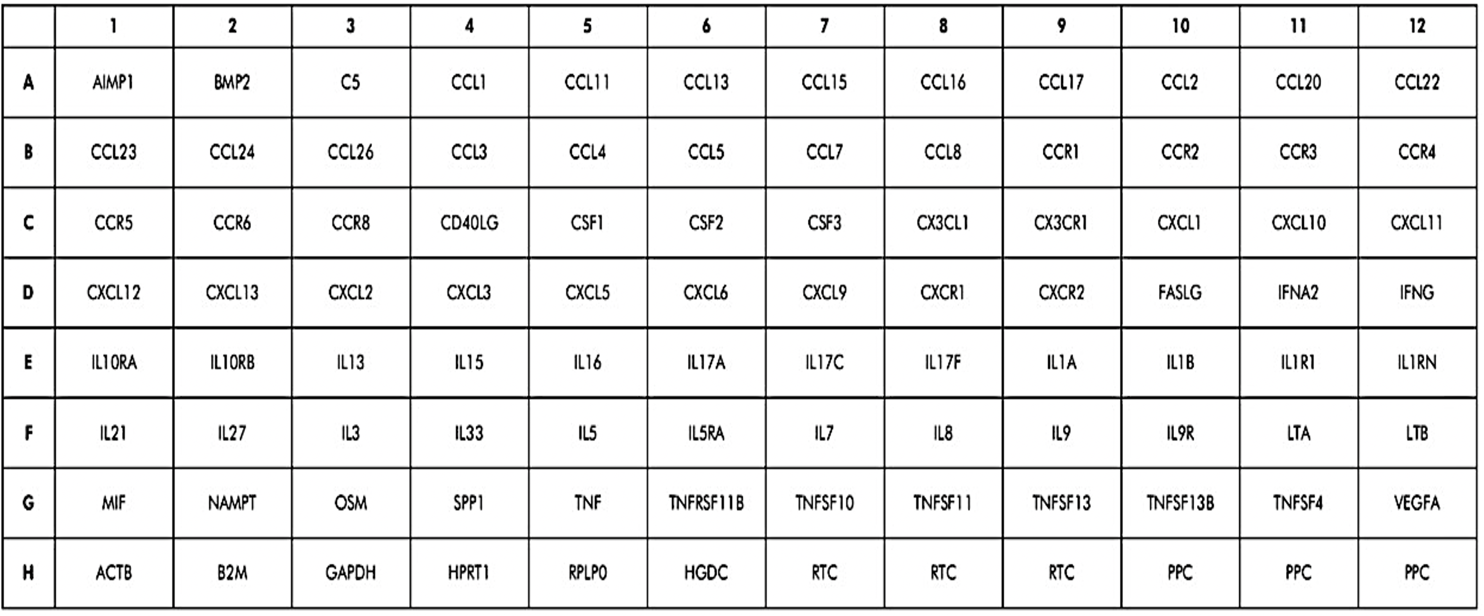


**Supplementary Figure 3:** Plate layout of the gene expression array depicting where the individual genes are located in each well.


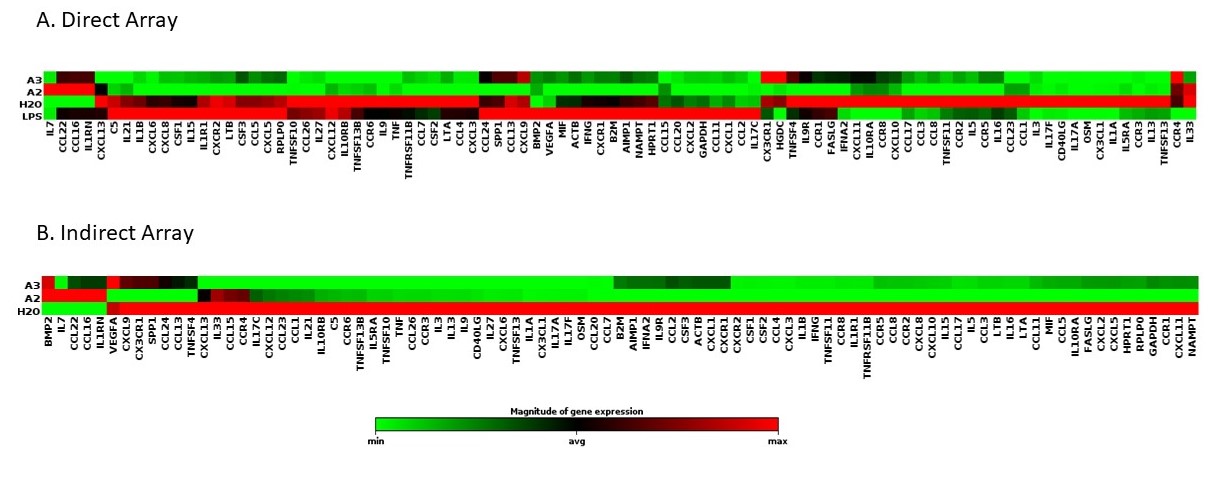


**Supplementary Figure 4: Gene expression profiles of A2 and A3.** A) Full plate gene expression array profile of TC-83-infected, A2/A3-treated U87MG cells. LPS was used as a positive control. B) Gene expression array profile of indirect inflammation (inflammation from uninfected cells) in U87MG cells.


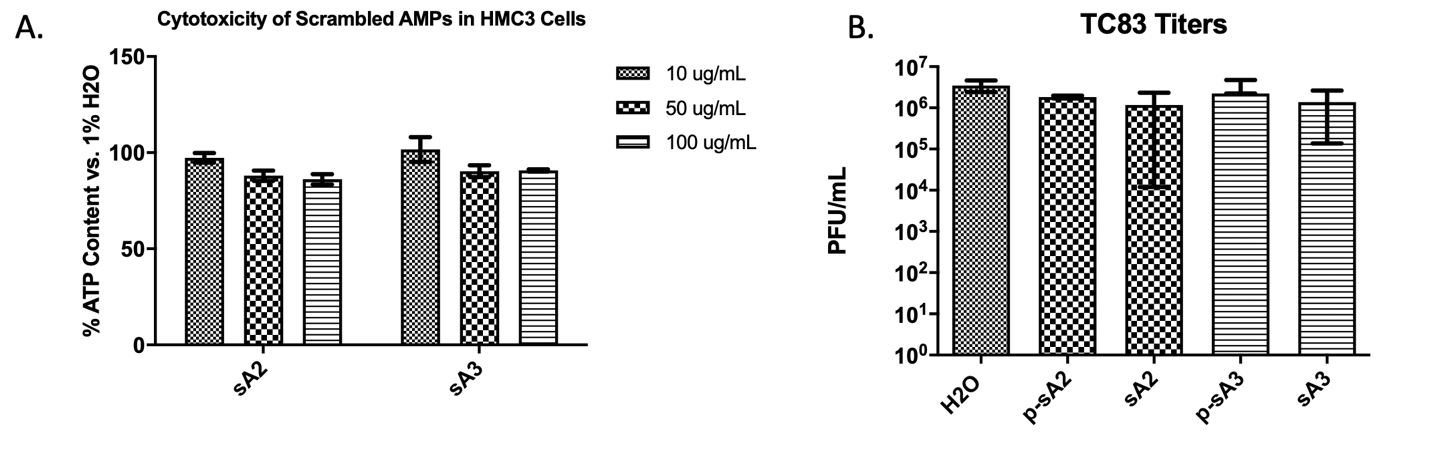


**Supplementary Figure 5: Efficacy of scrambled sequence controls of A2 and A3.** A) Cytotoxicity of scrambled A2 and A3 controls, reported as % vs. water control. B) Infectious titers of TC-83 in HMC3 cells following treatment with scrambled peptides. sA2 and sA3 represent respective scrambled peptide. p-sA2 and p-sA3 represent cells pre-treated with scrambled peptides for 2h prior to infection. The inhibition is not significant. n=3)

**Supplementary Table 1:**A2/A3-mediated gene expression regulation in infected cells

| A2 | | | | | | | |
| --- | --- | --- | --- | --- | --- | --- | --- |
| Gene | Fold Regulation | Gene | Fold Regulation | Gene | Fold Regulation | Gene | Fold Regulation |
| CCL16 | 2.19 | CCR2 | -7.36 | CXCR2 | -2.22 | IL9 | -3.51 |
| CCL22 | 2.06 | CCR3 | -2.71 | FASLG | -2.27 | IL9R | -2.48 |
| IL1RN | 2.19 | CCR5 | -3.23 | IFNA2 | -4.07 | LTA | -4.72 |
| IL7 | 9.88 | CCR6 | -5.15 | IL10RA | -3.22 | LTB | -3.31 |
| AIMP1 | -2.17 | CCR8 | -2.77 | IL10RB | -2.31 | NAMPT | -2.42 |
| C5 | -3.11 | CD40LG | -8.44 | IL13 | -4.71 | OSM | -9.67 |
| CCL1 | -2.21 | CSF1 | -2.23 | IL15 | -2.47 | SPP1 | -2.4 |
| CCL13 | -2.73 | CSF2 | -7.67 | IL16 | -2.42 | TNF | -9.95 |
| CCL17 | -2.71 | CSF3 | -8.01 | IL17A | -10.02 | TNFRSF11B | -4.24 |
| CCL20 | -2.91 | CX3CL1 | -25.15 | IL17F | -13.11 | TNFSF10 | -2.78 |
| CCL23 | -2.03 | CXCL1 | -2.79 | IL1A | -40.4 | TNFSF11 | -4.37 |
| CCL24 | -2.6 | CXCL10 | -2.98 | IL1B | -4.37 | TNFSF13 | -6.12 |
| CCL26 | -2.44 | CXCL11 | -4.17 | IL1R1 | -2.63 | TNFSF13B | -3.19 |
| CCL3 | -11.1 | CXCL2 | -2.97 | IL21 | -2.43 | TNFSF4 | -2.68 |
| CCL4 | -12.54 | CXCL3 | -8.92 | IL27 | -2.99 | B2M | -2.51 |
| CCL5 | -3.7 | CXCL5 | -3.43 | IL3 | -2.76 | GAPDH | -5.2 |
| CCL7 | -3.26 | CXCL6 | -3.46 | IL5 | -5.46 | HPRT1 | -2.53 |
| CCL8 | -6.18 | CXCL9 | -2.67 | IL5RA | -4.55 | RPLP0 | -2.88 |
| CCR1 | -2.78 | CXCR1 | -4.9 | CXCL8 | -4.74 |  |  |
| A3 | | | | | | | |
| C5 | -4.7 | CCR8 | -2.56 | IFNA2 | -2.17 | IL5RA | -6.83 |
| CCL1 | -3.46 | CD40LG | -13.61 | IL10RA | -2.36 | CXCL8 | -3.42 |
| CCL17 | -2.48 | CSF1 | -2.16 | IL10RB | -3.04 | IL9 | -4.24 |
| CCL20 | -2.88 | CSF2 | -6.65 | IL13 | -6.26 | LTA | -3.67 |
| CCL23 | -3.05 | CSF3 | -2.47 | IL15 | -2.18 | LTB | -2.77 |
| CCL26 | -2.76 | CX3CL1 | -40.25 | IL16 | -2.19 | OSM | -11.73 |
| CCL3 | -6.01 | CXCL10 | -2.5 | IL17A | -11.96 | TNF | -30.83 |
| CCL4 | -10.37 | CXCL11 | -2.45 | IL17C | -2.12 | TNFRSF11B | -3.73 |
| CCL5 | -2.56 | CXCL12 | -2.21 | IL17F | -17.44 | TNFSF10 | -3.34 |
| CCL7 | -3.29 | CXCL13 | -2.43 | IL1A | -75.02 | TNFSF11 | -3.96 |
| CCL8 | -3.92 | CXCL2 | -2.14 | IL1B | -3.92 | TNFSF13 | -7.83 |
| CCR1 | -2.01 | CXCL3 | -7.92 | IL1R1 | -2.45 | TNFSF13B | -4.76 |
| CCR2 | -4.3 | CXCL5 | -2.3 | IL21 | -4.12 | GAPDH | -2.65 |
| CCR3 | -3.13 | CXCL6 | -3.84 | IL27 | -3.21 | RPLP0 | -2.15 |
| CCR5 | -2.57 | CXCR1 | -2.13 | IL3 | -3.11 |  |  |
| CCR6 | -13.06 | CXCR2 | -2.17 | IL5 | -4.29 |  |  |

Genes from inflammatory array whose expression was regulated by a factor of 2 in infected cells.

**Supplementary Table 2:** A2/A3-mediated gene expression regulation in bystander cells

| A2 | | | |
| --- | --- | --- | --- |
| Gene | Fold Regulation | Gene | Fold Regulation |
| CCL16 | 4.62 | CX3CL1 | -2.85 |
| CCL22 | 4.62 | CXCL10 | -4.03 |
| CCL2 | -2.07 | CXCL11 | -7.19 |
| CCL5 | -3.09 | GAPDH | -9.26 |
| A3 | | | |
| CCL13 | 2.44 | IFNA2 | 2.06 |
| CCL16 | 3.4 | IL13 | 2.14 |
| CCL22 | 3.4 | IL17A | 2.61 |
| CCL23 | 2.82 | IL17C | 2.12 |
| CCL3 | 2.28 | IL21 | 2.21 |
| CCL7 | 2.34 | IL3 | 2.71 |
| CCL8 | 2.27 | IL33 | 3.21 |
| CCR2 | 3.03 | IL9 | 2.88 |
| CCR3 | 2.09 | IL9R | 2.56 |
| CCR5 | 2.2 | LTA | 2.46 |
| CCR6 | 2.3 | TNFSF11 | 2.33 |
| CX3CR1 | 2.38 | TNFSF13 | 2.31 |
| CXCL9 | 3.12 | CXCL11 | -3.38 |
| CXCR1 | 2.81 | CXCL6 | -2.9 |
| CXCR2 | 2.09 | GAPDH | -10.04 |
| FASLG | 2.15 |  |  |

Genes from inflammatory array whose expression was regulated by a factor of 2 in bystander cells.
